# The effects of dairy products containing *Lactobacillus bulgaricus* and *Streptococcus thermophilus* on bone turnover biomarkers and bone mineral density: a pilot trial study

**DOI:** 10.3389/fnut.2026.1785177

**Published:** 2026-03-05

**Authors:** Narisa Rueangsri, Uraiporn Booranasuksakul, Natthapaninee Thanomsridetchai, Palatip Chutoam, Lukkamol Prapkree, Alongkote Singhato

**Affiliations:** 1 Nutrition and Dietetics Division, Faculty of Allied Health Sciences, Burapha University, Sansook, Mueang, Chonburi, Thailand; 2 Medical Technology Division, Faculty of Allied Health Sciences, Burapha University, Sansook, Mueang, Chonburi, Thailand; 3 Sodexo at the University of Kansas Health System, Olathe, KS, United States

**Keywords:** bone mineral density, bone turnover, dairy product, DEXA, probiotics

## Abstract

**Background/objectives:**

Low bone mineral density is a major public health concern that negatively affects quality of life. Dairy products fermented with *Lactobacillus bulgaricus* and *Streptococcus thermophilus* have been suggested to promote gut health, enhance calcium absorption, and suppress inflammatory cytokines related to bone turnover. However, the benefits of these fermented dairy products on bone health remain unclear. This study aimed to compare the effects of consuming dairy products with and without *Lactobacillus bulgaricus* and *Streptococcus thermophilus* on bone turnover biomarkers and bone mineral density.

**Methods:**

A randomized controlled pilot trial was conducted in a total of 40 female participants, who were allocated to either a control group (*n* = 20) receiving full-fat milk or an intervention group (*n* = 20) receiving yogurt fermented with *Lactobacillus bulgaricus* and *Streptococcus thermophilus*. Both groups consumed products providing an equivalent calcium intake of 400 mg/day for 12 weeks. Blood samples were collected, and body composition and bone mineral density were assessed.

**Results:**

At the endpoint, serum calcium levels in the intervention group were significantly higher than those in the control group (*p* < 0.05). Alkaline phosphatase and parathyroid hormone levels were significantly reduced from baseline in both groups (*p* < 0.05). Bone mineral density at the right arm, right leg, and pelvis was significantly increased from baseline in the intervention group (*p* < 0.05); however, no significant between-group differences in bone mineral density were observed at the endpoint.

**Conclusion:**

Although within-group improvements in bone mineral density were observed, no significant differences between groups were detected. These findings should be interpreted with caution due to the pilot nature of the study, and larger, adequately powered randomized controlled trials are warranted to confirm the effects on bone mineral density.

**Clinical Trial Registration:**

Identifier: TCTR20250807006.

## Introduction

Loss of bone mineral density (BMD), which leads to bone fragility and osteoporosis, is a major public health problem that is increasingly prevalent worldwide (1, 2). This condition primarily results from an imbalance between bone resorption and bone formation, often exacerbated by inadequate intake of key nutrients essential for bone health from early adulthood, particularly calcium. Low BMD substantially impairs quality of life, as it is commonly associated with chronic pain, reduced mobility, and an increased risk of fractures (3). Epidemiological data indicate that bone-related disorders are highly prevalent across many regions. In the United States, approximately 10 million individuals aged over 50 years are affected by osteoporosis (4), while in Japan the estimated number reaches 15 million (5). In Thailand, the prevalence of bone fragility and osteoporosis has steadily increased, largely due to population aging and persistently insufficient calcium intake (6). A previous study among Thai older adults residing in long-term care facilities reported that up to 89% exhibited bone fragility, with particularly high prevalence among postmenopausal women (7).

Insufficient calcium intake is a well-established determinant of hypocalcemia (8), which has been consistently associated with reduced BMD (9). National dietary surveys indicate that average calcium intake among Thai individuals is approximately 300 mg/day, substantially lower than the recommended intake of 900 mg/day (10). This inadequacy is partly attributable to dietary habits and cultural practices that limit the consumption of milk and dairy products, which are among the most important dietary sources of bioavailable calcium. Importantly, in young adults, behavioral factors such as perceived lactose intolerance and voluntary avoidance of dairy products may further compromise calcium intake. Recent evidence has demonstrated that self-reported lactose intolerance and avoidance of milk and dairy products are independently associated with lower BMD in young adults, even after adjustment for lifestyle-related factors (11). These findings suggest that not only calcium content, but also the form and tolerability of dairy products, may influence bone health during early adulthood.

In addition to the calcium on bone health, previous studies have also highlighted the potential benefits of foods containing probiotics, which promote various aspects of health, including immune function, bowel movement, and improved nutrient absorption, especially enhanced absorption of calcium and vitamin D, which play crucial roles in bone formation (12, 13). Yogurt, a calcium-rich food produced through microbial fermentation, represents a widely consumed vehicle for probiotic delivery. In Thailand, *Lactobacillus bulgaricus* and *Streptococcus thermophilus* are the principal bacterial strains used in yogurt fermentation and are recognized as beneficial microorganisms. Mechanistically, probiotics may influence bone metabolism through the gut–bone axis by modulating intestinal microbiota composition, reducing gut-derived inflammation, and improving mineral bioavailability. Experimental and clinical studies have suggested that intake of *Lactobacillus bulgaricus* and *Streptococcus thermophilus* can suppress inflammatory responses in intestinal cells (14), enhance the absorption of minerals and micronutrients such as calcium, vitamin B12, folate, iron, and zinc (15), and reduce systemic inflammatory markers associated with increased bone turnover (16). Furthermore, these probiotic strains have been shown to promote gut health, improve mineral absorption, and suppress pro-inflammatory mediators involved in bone resorption (17). Collectively, these mechanisms provide a biological rationale for the potential role of probiotic-fermented dairy products in supporting bone health. Although the well-recognized burden of low BMD and the reported benefits of calcium intake and probiotics—particularly *Lactobacillus bulgaricus* and *Streptococcus thermophilus*, evidence regarding their combined effects on bone turnover and BMD in humans remains limited. Moreover, the potential advantage of fermented dairy products as a more tolerable form of calcium intake, especially among young adults who may avoid conventional milk, has not been sufficiently explored. Therefore, this randomized controlled pilot trial aimed to investigate the effects of consuming dairy products fermented with *Lactobacillus bulgaricus* and *Streptococcus thermophilus* on changes in bone turnover biomarkers and bone mineral density, compared with conventional dairy products without probiotics.

## Methods

### Study tools

#### Fermented dairy product (yogurt) used in this study

The yogurt product selected for this study was full-fat plain yogurt (Dutchie^®^), contains 20 billion CFU of microorganisms per serving, a brand that is widely available in the local Thai markets and produced using *Lactobacillus bulgaricus* and *Streptococcus thermophilus* as starter cultures in the fermentation process. The full-fat milk product was pasteurized whole milk (CP-Meiji^®^), which is also commonly available in the local Thai markets. The nutrient and calcium contents of both products were calculated based on the nutrition information provided on the product labels.

#### The baseline and 3 days-dietary record questionnaire

A validated both closed-ended and opened-ended questionnaire was developed to collect baseline information from the participants, such as age, sex, and educational level, as well as data obtained from body composition measurements and the analysis of bone turnover biomarkers from blood sample. In addition, participants were asked to complete a 3-day dietary record after enrolling in the study and receiving instructions on portion size estimation. The dietary record included 2 weekdays and 1 weekend day, and was completed during the final week of the study (18). The completed dietary records were used for calculation of energy and nutrient intake, including total energy intake, energy distribution, and daily calcium intake using the INMUCAL-Nutrient software, version 4.0, developed by the Institute of Nutrition, Mahidol University, together with data from the United States Department of Agriculture (USDA) food composition database.

#### Measurement of bone mineral density and body composition analysis

Body composition was assessed using the InBody270 (InBody Co., Seoul, South Korea) and the BOD POD system (COSMED Inc., Rome, Italy). These instruments were used to analyze body composition parameters, such as percentage body fat, muscle mass, and body fluid percentage. Bone mineral density was measured at several skeletal sites, including the right and left arm, lumbar spine, rip, pelvis, etc. Measurements were performed using dual-energy X-ray absorptiometry (DEXA) with the Medix DR system (DMS Imaging Inc., Gallargues-le-Montueux, France). The DEXA system was certified for radiation safety by the Thailand Department of Medical Sciences, and calibration was conducted prior to each measurement to ensure data reliability.

### Participants

This randomized controlled pilot trial recruited a total of 40 participants, based on an a priori sample size estimation using G^*^Power, who were randomly assigned to either the intervention group (*n* = 20) or the control group (*n* = 20). The inclusion criteria were Thai adult women aged 20–40 years, selected to minimize potential hormonal confounding factors and because this age range has the same amount of daily calcium requirements. The exclusion criteria included the presence of any chronic medical conditions, particularly liver-related diseases; gastrointestinal disorders; a history of cow's milk protein allergy or lactose intolerance; current use of herbal products or dietary supplements; pregnancy or lactation; a history of bone fractures due to any cause; and the use of medications known to affect bone mineral density, such as bisphosphonates or vitamin D supplements.

### Study procedures

The participants who met the inclusion and exclusion criteria were scheduled to attend an appointment and invited at a designated room within the Faculty of Allied Health Sciences, Burapha University, Thailand, where written informed consent was obtained. Participants were then randomly assigned to either the intervention group or the control group, with 20 participants in each group. All participants received instructions on the Thai food exchange list to support accurate completion of a 3-day dietary record, which was to be submitted during the final week of the study. The dietary record included 2 weekdays and 1 weekend day.

Baseline assessments were then conducted at the Clinical Nutrition Laboratory, Faculty of Allied Health Sciences, Burapha University. These assessments included measurements of body composition using the InBody270 and BOD POD devices, as well as bone mineral density assessment using DEXA (DEXA measurements were performed by assessors who were blinded to group allocation). Following these measurements, venous blood samples (10 mL) were collected from the antecubital vein by a medical technologist to determine serum levels of alkaline phosphatase (ALP)—related marker due to its widespread availability and established use in clinical and epidemiological studies, parathyroid hormone (PTH), osteocalcin (OC), and total serum calcium.

After completion of the baseline procedures, participants in the intervention group received full-fat plain yogurt (Dutchie^®^), fermented with *Lactobacillus bulgaricus* and *Streptococcus thermophilus*. The daily intake was set at 200 g of yogurt consumed after lunch, providing approximately 400 mg of calcium per day, which corresponds to 50% of the recommended daily calcium intake for Thai adults aged 20–40 years. This amount was based on previous findings indicating that a daily calcium intake of at least 400 mg is the minimum level associated with significant improvements in height and bone mineral density (19, 20). Participants were instructed and shown how to accurately measure the required portion size. Throughout the 12-week intervention period, participants were asked to refrain from consuming milk, yogurt, or other dairy products in addition to the study product.

Participants in the control group received pasteurized full-fat plain milk (CP-Meiji^®^) and were instructed to consume 400 mL per day (two small bottles) after meals, providing an equivalent calcium intake of 400 mg per day, for the same 12-week period. Similar to the intervention group, control participants were asked to avoid consuming other dairy products during the study. Adherence to yogurt and milk consumption in both groups was monitored daily via the LINE application.

At the end of the 12-week intervention, participants from both groups were invited back to the Faculty of Allied Health Sciences, Burapha University. They submitted their completed 3-day dietary records and underwent follow-up assessments, including body composition measurement, bone mineral density measurement, and venous blood collection (10 mL) for analysis of ALP, PTH, OC, and total serum calcium. All collected data were subsequently used for statistical analysis.

## Statistical analyses

Baseline characteristics such as sex and educational level were reported as percentages. Other baseline and study-related variables, including age, weekly exercise duration, levels of bone turnover biomarkers (ALP, PTH, OC, and total serum calcium), body composition parameters (e.g., body weight, muscle mass, fat mass, basal metabolic rate), bone mineral density values (T-scores and BMD values), and nutrient intake were presented as mean ± standard deviation (mean ± SD). Differences in mean values between the intervention and control groups, both before and after completion of the study, were analyzed using independent *t*-tests. Within-group comparisons before and after the intervention were performed using paired *t*-tests. Statistical analyses were conducted using Predictive Analytics Software Statistics (SPSS Inc., Chicago, IL, United States), version 26.0. A *p*-value of < 0.05 was considered statistically significant.

## Results

### Background of the participants

All of the participants were Thai females, with a mean age of 21.85 years for the control group and 22.15 years for the intervention group. Participants in both groups were within the normal BMI range, and most of them (90% for the control group and 95% for the intervention group) held a bachelor's degree. The length of weekly exercise was 52.25 min for the control group and 62.25 min for the intervention group. Statistical analyses found no significant differences in background data between groups (Table 1).

**Table 1:** Background characteristic of study participants.

**Characteristic**	**Controlled group (*n* = 20)**	**Intervention group (*n* = 20)**	***p* value**
Age (year), mean (SD)	21.85 (1.49)	22.15 (2.20)	0.67^a^
BMI, mean (SD)	21.35 (2.64)	22.05 (2.62)	0.53^a^
**Education**			
- Bachelor degree, *n* (%)	18 (90)	19 (95)	0.50^b^
- Graduate degree, *n* (%)	2 (10)	1 (5)	
Length of weekly exercise (minute), mean (SD)	52.25 (68.46)	62.25 (77.56)	0.69^a^

^a^Paired-samples *t*-test, ^b^Fisher exact two-sided test.

### Biomarker of bone turnover and bone mineral density

Effects of dairy products containing *Lactobacillus bulgaricus* and *Streptococcus thermophilus* on bone health were determined by biomarkers of participants and bone mineral density measurements. The results revealed that participants in the intervention group had significantly higher serum calcium than the control group at the endpoint (9.83 vs. 9.28 mg/dL; *p* < 0.05). In addition, the results found that the intervention group had significantly lower ALP and PTH levels at the endpoint when compared to baseline (56.95 vs. 73.10 for ALP; *p* < 0.05 and 36.66 vs. 41.01 for PTH; *p* < 0.05). Whereas participants in the control group indicated lower ALP only at the endpoint when compared to baseline (60.90 vs. 68.30; *p* < 0.05).

For bone mineral density, the results revealed that there was no significant difference between groups at the endpoint. However, participants in the intervention group were found to have significantly higher T-scores at the endpoint when compared to baseline for the right arm (1.32 vs. 0.94; *p* < 0.05), pelvis (−0.48 vs. −1.38; *p* < 0.05), and right leg (0.47 vs. −0.05; *p* < 0.05) (Table 2).

**Table 2:** Biomarkers of bone turnover and bone mineral density.

**Biomarkers of bone turnover and various parts of bone measurement**	**Baseline**			**Endpoint**		
	**Controlled**	**Intervention**	***p*** **value**	**Controlled**	**Intervention**	***p*** **value**
ALP (IU/L), mean (SD)	68.30 (16.10)	73.10 (16.10)	0.34	60.90 (11.65)^∧^	56.95 (9.53)^∧^	0.24
PTH (pg/ml), mean (SD)	37.15 (13.99)	41.01 (13.03)	0.37	35.81 (12.90)	36.66 (10.74)^∧^	0.82
OC (ng/ml), mean (SD)	30.20 (9.86)	29.10 (8.82)	0.71	31.30 (6.75)	32.65 (5.81)	0.50
serum calcium (mg/dL), mean (SD)	8.87 (0.39)	8.96 (0.45)	0.51	9.28 (0.43)^∧^	9.83 (0.33)^∧^	< 0.05^*^
T-score of left arm, mean (SD)	0.99 (1.03)	0.78 (0.86)	0.49	0.93 (0.98)	0.81 (0.80)	0.67
BMD of left arm (in g/cm^2^), mean (SD)	0.89 (0.09)	0.87 (0.07)	0.47	0.88 (0.08)	0.87 (0.07)	0.65
T-score of right arm, mean (SD)	1.24 (1.11)	0.94 (0.86)	0.34	1.28 (1.15)	1.32 (0.67)^∧^	0.89
BMD of right arm (in g/cm^2^), mean (SD)	0.91 (0.10)	0.88 (0.07)	0.37	0.91 (0.10)	0.89 (0.07)	0.45
T-score of left rib, mean (SD)	−1.75 (1.01)	−1.86 (1.14)	0.73	−1.82 (0.90)	−1.82 (1.17)	0.98
BMD of left rib (in g/cm^2^), mean (SD)	0.85 (0.12)	0.84 (0.13)	0.75	0.84 (0.10)	0.84 (0.13)	0.99
T-score of right rib, mean (SD)	−1.85 (0.86)	−1.83 (1.08)	0.94	−1.88 (0.85)	−1.73 (1.11)	0.63
BMD of right rib (in g/cm^2^), mean (SD)	0.84 (0.10)	0.84 (0.12)	0.98	0.84 (0.10)	0.85 (0.13)	0.63
T-score of T spine, mean (SD)	0.85 (1.2)	0.85 (1.0)	0.99	0.83 (1.19)	0.82 (1.10)	0.97
BMD of T spine (in g/cm^2^), mean (SD)	0.98 (0.12)	0.98 (0.10)	0.97	0.98 (0.11)	0.98 (0.11)	0.96
T-score of L spine, mean (SD)	−1.07 (1.18)	−1.08 (0.76)	0.96	−1.00 (0.86)	−1.05 (0.64)	0.85
BMD of L spine (in g/cm^2^), mean (SD)	1.04 (0.15)	1.04 (0.10)	0.95	1.05 (0.11)	1.05 (0.08)	0.87
T-score of pelvis, mean (SD)	−1.15 (0.68)	−1.38 (0.66)	0.27	−1.05 (0.62)	−0.48 (1.41)^∧^	0.10
BMD of L pelvis (in g/cm^2^), mean (SD)	1.04 (0.09)	1.01 (0.08)	0.28	1.05 (0.08)	1.02 (0.08)	0.21
T-score of left leg, mean (SD)	0.12 (0.82)	−0.05 (0.76)	0.49	0.16 (0.81)	−0.06 (0.75)	0.35
BMD of left leg (in g/cm^2^), mean (SD)	1.04 (0.09)	1.02 (0.08)	0.51	1.05 (0.09)	1.02 (0.08)	0.38
T-score of right leg, mean (SD)	0.23 (0.86)	−0.05 (0.76)	0.28	0.21 (0.85)	0.47 (0.56)^∧^	0.26
BMD of right leg (in g/cm^2^), mean (SD)	1.05 (0.09)	1.02 (0.08)	0.28	1.05 (0.09)	1.03 (0.08)	0.43
T-score of total, mean (SD)	−0.35 (0.78)	−0.51 (0.68)	0.48	−0.36 (0.74)	−0.50 (0.69)	0.54
BMD of total (in g/cm^2^), mean (SD)	0.98 (0.08)	0.96 (0.07)	0.45	0.98 (0.08)	0.96 (0.07)	0.51

^*^significant difference between groups at *p* < 0.05.

∧significant difference within group compared with baseline at *p* < 0.05.

### Body composition

Regarding body composition analysis, the components including %body fat, total body fluid, muscle mass, mineral weight, basal metabolic rate, total energy requirement, and thoracic gas volume were determined. The results showed that %body fat significantly increased within both groups at the endpoint (for controlled group 20.67 vs. 25.10; *p* < 0.05, and for intervention group 22.96 vs. 26.46; *p* < 0.05). However, no significant differences were found for other parameters either between groups or within groups when comparing baseline and endpoint (Table 3).

**Table 3:** Change of body composition at baseline and endpoint.

**Body composition measurement**	**Baseline**			**Endpoint**		
	**Controlled**	**Intervention**	***p*** **value**	**Controlled**	**Intervention**	***p*** **value**
%body fat, mean (SD)	20.67 (9.95)	22.96 (10.33)	0.48	25.10 (5.72)^∧^	26.46 (6.60)^∧^	0.49
Total body fluid (L), mean (SD)	26.95 (2.63)	27.24 (3.35)	0.76	27.03 (2.74)	27.37 (3.17)	0.71
Muscle mass (kg), mean (SD)	43.09 (4.81)	42.50 (4.88)	0.70	43.14 (4.88)	43.97 (4.64)	0.58
Mineral weight (kg), mean (SD)	2.64 (0.29)	2.68 (0.32)	0.69	2.66 (0.30)	2.72 (0.30)	0.52
Basal metabolic rate (kcal), mean (SD)	1,165.10 (78.54)	1,173.70 (99.23)	0.76	1,168.05 (81.41)	1,178.05 (93.98)	0.72
Total energy requirement (kcal), mean (SD)	1,795.05 (304.74)	1,793.55 (269.67)	0.98	1,821.85 (257.67)	1,889.20 (238.78)	0.39
Thoracic gas volume (L), mean (SD)	2.98 (0.17)	3.06 (0.25)	0.26	2.98 (0.18)	3.05 (0.25)	0.33

^∧^significant difference within group compared with baseline at *p* < 0.05.

### Participants' dietary habits

Participants' dietary habits were assessed using the submitted food records. The results revealed that there were no significant differences in all nutrients consumed among participants in both groups, such as the percentage of energy derived from macronutrients and trace minerals (Table 4).

**Table 4:** Dietary habits of participants.

**Dietary consumed**	**Controlled group (*n* = 20)**	**Intervention group (*n* = 20)**	***p* value**
Total kcal intake (kcal), mean (SD)	2,070.55 (311.43)	2,065.95 (257.08)	0.95
%kcal distributed from carbohydrate, mean (SD)	54.55 (5.16)	54.30 (5.00)	0.87
%kcal distributed from protein, mean (SD)	16.25 (4.57)	16.75 (4.36)	0.64
%kcal distributed from fat, mean (SD)	29.20 (6.71)	28.95 (6.06)	0.88
Daily sodium intake (mg), mean (SD)	3,397.95 (980.87)	3,312.60 (892.70)	0.27
Daily calcium intake (mg), mean (SD)	753.10 (135.96)	748.45 (106.98)	0.90
Daily vitamin D intake (IU), mean (SD)	485.65 (48.32)	474.80 (73.89)	0.62
Daily magnesium intake (mg), mean (SD)	303.30 (44.44)	293.25 (39.54)	0.46
Daily phosphorus intake (mg), mean (SD)	688.55 (67.93)	672.90 (55.85)	0.40

## Discussion

Consuming an adequate amount of calcium is widely accepted as a key factor in promoting bone health and reducing the risk of bone-related complications later in life. Overall, this study indicates that the intake of dairy products which are good sources of calcium both with and without *Lactobacillus bulgaricus* and *Streptococcus thermophilus* is beneficial for improving bone mineral density among participants in some positions. However, the results showed that serum calcium levels among participants in the intervention group were higher than those in the control group at the endpoint. This finding supports previous studies suggesting that *Lactobacillus bulgaricus* and *Streptococcus thermophilus* enhance calcium absorption, primarily by lowering gut pH through lactic acid production, which increases calcium solubility and bioavailability (21). Moreover, *Lactobacillus bulgaricus* and *Streptococcus thermophilus* can stimulate the production of short-chain fatty acids (SCFAs). These compounds not only further reduce intestinal pH but also create a favorable environment for calcium absorption, particularly in the large intestine (22). This mechanism may also explain the lower PTH levels observed at the endpoint in the intervention group compared with baseline. When blood calcium levels rise, the parathyroid glands detect this change via calcium-sensing receptors (CaSR) located on the surface of parathyroid cells. Elevated calcium activates these receptors, triggering intracellular signaling pathways that inhibit both the synthesis and secretion of PTH (23). Consequently, PTH levels decrease, reducing its calcium-raising effects on bone, kidneys, and the intestine (24). For ALP, a significant reduction at the endpoint was observed in both groups. This may be explained by the regular intake food source of calcium over several months, which helps maintain stable and sufficient blood calcium levels. As a result, stimulation of the parathyroid glands decreases, leading to lower PTH secretion. With reduced PTH activity, bone resorption declines and overall bone turnover slows. As bone remodeling activity decreases, osteoblasts produce less ALP, resulting in lower serum ALP levels (25–27).

Although statistically significant within-group increases in bone mineral density were observed at selected skeletal sites in the intervention group, the magnitude of these changes should be interpreted cautiously. In young adult women, bone mineral density is generally stable, and small short-term changes may not necessarily translate into clinically meaningful improvements in fracture risk (28). Currently, there is no universally accepted threshold defining a clinically significant change in bone mineral density for this age group; therefore, the observed changes primarily suggest potential biological responsiveness rather than definitive clinical benefit. In addition, bone mineral density in the right arm, pelvis, and right leg was found to be increased among participants in the intervention group compared with baseline. This finding may be attributed to the interaction between calcium availability and mechanical loading. Bone tissue adapts to habitual stress, and regions subjected to greater mechanical forces tend to exhibit enhanced mineral deposition. As most participants are likely to be right-side dominant, the dominant limbs and pelvis may experience higher levels of daily loading through routine activities and weight-bearing movements. In this context, calcium intake provides the necessary mineral substrate, while mechanical loading determines the sites of bone formation (29, 30). The mechanisms of *Lactobacillus bulgaricus* and *Streptococcus thermophilus* have been described in previous study, which indicate that these probiotic strains play a significant role in the bone remodeling process by maintaining the delicate balance between bone-forming cells (osteoblasts) and bone-resorbing cells (osteoclasts). These probiotics may affect bone metabolism through multiple pathways, including modulation of gut microbiota, enhancement of mineral absorption, and reduction of systemic inflammation, all of which contribute to the regulation of bone turnover. Proteins and various minerals are essential components of bone tissue. Dairy products, particularly fermented dairy products (yogurt), are important dietary sources of high-quality proteins and bioavailable minerals that are required for the formation and maintenance of healthy bone structure (31).

The Body composition measurements revealed that, at the endpoint, the percentage of body fat had increased from baseline in both groups. Although dietary data indicated no significant differences in overall nutrient intake between groups, the consumption of full-fat milk combined with low levels of physical activity among participants (as shown by the short duration of weekly exercise) may have contributed to the increase in body fat percentage (32–34). Interestingly, despite receiving dairy products that are rich sources of calcium, calcium intake among participants in both groups did not reach the recommended level of 900 mg per day for adults (35). Nevertheless, this level of calcium intake may still be sufficient to reduce bone turnover, as suggested by previous studies (36, 37). Vitamin D consumed was also below the recommended level among participants at 600 IU per day (38). Therefore, these findings highlight the importance of promoting sun exposure to support endogenous vitamin D synthesis, which plays a crucial role in enhancing calcium absorption and bone formation (39). This trial study has several limitations. It was conducted with a relatively small sample size and over a short intervention period of 12 weeks. Hence, future studies should be conducted in a larger population and over a longer duration, with the inclusion of additional blood biomarkers of bone turnover and bone formation, such as Bone-specific alkaline phosphatase (BSAP), C-terminal telopeptide of type I collagen (CTX), and procollagen type I N-terminal propeptide (P1NP) (40, 41). Since probiotics in fermented dairy products can generate anti-inflammatory substances in the human gastrointestinal system and within the body, they may influence parameters related to bone turnover (42).

## Conclusion

In conclusion, the findings of this study suggest that the consumption of dairy products fermented with *Lactobacillus bulgaricus* and *Streptococcus thermophilus* may provide additional benefits for bone health beyond calcium intake alone. Although no significant between-group differences in bone mineral density were observed at the endpoint, significant within-group increases in T-scores at the right arm, pelvis, and right leg were detected in the intervention group. In young adult women, bone mineral density is generally stable, and such within-group changes may represent early biological or adaptive responses to probiotic-fermented dairy intake rather than clinically definitive improvements. The lack of between-group differences may be explained by the limited sample size, inter-individual variability, and the relatively short intervention period, which together reduce the ability to detect modest treatment effects. These findings should therefore be interpreted cautiously and viewed as hypothesis-generating in the context of this pilot study.

## Data Availability

The datasets presented in this study can be found in online repositories. The names of the repository/repositories and accession number(s) can be found in the article/supplementary material.
